# 

**Published:** 1859-06

**Authors:** 


### DENTAL REGISTER

### OF THE WEST.

EDITED BY

J. TAFT AND GEO. WATT.

June, 1859.

vol 12—4

Published Quarterly at $3 Per Annum.

CINCINNATI:

J. T. TOLAND, PUBLISHER AND PROPRIETOR.

1859.

